# Supplementary material for: Hybrid Models and Biological Model Reduction with PyDSTool
Source: PLoS Comput Biol. 2012 Aug 9;8(8):e1002628. doi: 10.1371/journal.pcbi.1002628 (PMC3415397; doi:10.1371/journal.pcbi.1002628)
Supplement: Text S4 — Complete source code for the PyDSTool package (version 0.88.120504). Includes API documentation and help files linking to web pages. This file is identical to the current public release on Sourceforge.net. (ZIP) [file pcbi.1002628.s004.zip › PyDSTool/html/PyDSTool.scipy_ode.vode-class.html]

xml version="1.0" encoding="ascii"?


PyDSTool.scipy\_ode.vode


| Home | Trees | Indices | Help | PyDSTool |
| --- | --- | --- | --- | --- |

Package PyDSTool ::
Module scipy\_ode ::
Class vode

[hide private]

[frames] | no frames]

# Class vode

source code

```
    object --+    
             |    
IntegratorBase --+
                 |
                vode
```

---


|  |  |
| --- | --- |
| Instance Methods | [hide private] |

|  |  |
| --- | --- |
| \_\_init\_\_(self, method=`'``adams``'`, with\_jacobian=0, rtol=1e-06, atol=1e-12, lband=None, uband=None, order=12, nsteps=500, max\_step=0.0, min\_step=0.0, first\_step=0.0)  x.\_\_init\_\_(...) initializes x; see x.\_\_class\_\_.\_\_doc\_\_ for signature | source code |

|  |  |
| --- | --- |
| reset(self, n, has\_jac)  Prepare integrator for call: allocate memory, set flags, etc. | source code |

|  |  |
| --- | --- |
| run(self, \*args)  Integrate from t=t0 to t=t1 using y0 as an initial condition. | source code |

|  |  |
| --- | --- |
| step(self, \*args)  Make one integration step and return (y1,t1). | source code |

|  |  |
| --- | --- |
| run\_relax(self, \*args)  Integrate from t=t0 to t>=t1 and return (y1,t). | source code |

**Inherited from `object`**:
`__delattr__`,
`__getattribute__`,
`__hash__`,
`__new__`,
`__reduce__`,
`__reduce_ex__`,
`__repr__`,
`__setattr__`,
`__str__`


|  |  |
| --- | --- |
| Class Variables | [hide private] |

\_vode  
This module 'vode' is auto-generated with f2py (version:2\_5540).

runner = `getattr(_vode, 'dvode', None)`

messages = `{-6: 'Error weight became zero during problem. (Sol...`

supports\_run\_relax = `1`

supports\_step = `1`

**Inherited from `IntegratorBase`**:
`integrator_classes`,
`success`


|  |  |
| --- | --- |
| Properties | [hide private] |

**Inherited from `object`**:
`__class__`


|  |  |
| --- | --- |
| Method Details | [hide private] |

|  |  |
| --- | --- |
| \_\_init\_\_(self, method=`'``adams``'`, with\_jacobian=0, rtol=1e-06, atol=1e-12, lband=None, uband=None, order=12, nsteps=500, max\_step=0.0, min\_step=0.0, first\_step=0.0)  *(Constructor)* | source code |

x.\_\_init\_\_(...) initializes x; see x.\_\_class\_\_.\_\_doc\_\_ for
signature

Overrides: object.\_\_init\_\_: *(inherited documentation)*

|  |  |
| --- | --- |
| reset(self, n, has\_jac) | source code |

Prepare integrator for call: allocate memory, set flags, etc. n -
number of equations. has\_jac - if user has supplied function for
evaluating Jacobian.

Overrides: IntegratorBase.reset: *(inherited documentation)*

|  |  |
| --- | --- |
| run(self, \*args) | source code |

Integrate from t=t0 to t=t1 using y0 as an initial condition. Return
2-tuple (y1,t1) where y1 is the result and t=t1 defines the stoppage
coordinate of the result.

Overrides: IntegratorBase.run: *(inherited documentation)*

|  |  |
| --- | --- |
| step(self, \*args) | source code |

Make one integration step and return (y1,t1).

Overrides: IntegratorBase.step: *(inherited documentation)*

|  |  |
| --- | --- |
| run\_relax(self, \*args) | source code |

Integrate from t=t0 to t>=t1 and return (y1,t).

Overrides: IntegratorBase.run\_relax: *(inherited documentation)*

  


|  |  |
| --- | --- |
| Class Variable Details | [hide private] |

### \_vode

```
This module 'vode' is auto-generated with f2py (version:2_5540).
Functions:
  y,t,istate = dvode(f,jac,y,t,tout,rtol,atol,itask,istate,rwork,iwork,mf,f_extra_args=(),jac_extra_args=(),overwrite_y=0)
  y,t,istate = zvode(f,jac,y,t,tout,rtol,atol,itask,istate,zwork,rwork,iwork,mf,f_extra_args=(),jac_extra_args=(),overwrite_y=0)
.
```

Value:
:   scipy.integrate.vode

### messages

Value:
:   ```
    {-6: 'Error weight became zero during problem. (Solution component i v
    anished, and ATOL or ATOL(i) = 0.)',
     -5: 'Repeated convergence failures. (Perhaps bad Jacobian supplied or
     wrong choice of MF or tolerances.)',
     -4: 'Repeated error test failures. (Check all input.)',
     -3: 'Illegal input detected. (See printed message.)',
     -2: 'Excess accuracy requested. (Tolerances too small.)',
     -1: 'Excess work done on this call. (Perhaps wrong MF.)'}
    ```

  


| Home | Trees | Indices | Help | PyDSTool |
| --- | --- | --- | --- | --- |

|  |  |
| --- | --- |
| Generated by Epydoc 3.0.1 on Fri May 4 15:24:10 2012 | http://epydoc.sourceforge.net |
